# Challenges in interpreting individual-level changes in health-related quality of life in patients with glioma using minimally important differences (MIDs) and a 4-point Likert scale

**DOI:** 10.1007/s11136-025-04029-3

**Published:** 2025-08-05

**Authors:** Ogechukwu A. Asogwa, Johan A. F. Koekkoek, Marthe C. M. Peeters, Hanneke Zwinkels, Maaike J. Vos, Linda Dirven, Martin J. B. Taphoorn

**Affiliations:** 1 Department of Neurology, Leiden University Medical Center, Leiden, The Netherlands; 2 Department of Neurology, Haaglanden Medical Center, The Hague, The Netherlands

**Keywords:** Brain tumor, Glioma, Minimally important differences (MIDs), Health-related quality of life (HRQoL), Response Likert scale, Patients reported outcome (PRO)

## Abstract

**Purpose:**

Interpretation of changes on the individual level is often based on minimally important differences (MIDs) developed on the group level. We investigated the impact of applying different group-level MIDs (anchor-based and 10-point MIDs) to determine health-related quality of life (HRQoL) changes in glioma patients. We further explored directions and magnitudes of these changes and their relationship to response formats and types of scale.

**Methods:**

We included 92 glioma patients at least 18 years old from a previously conducted randomized prospective study. We calculated changes in HRQoL (EORTC QLQ-C30 and QLQ-BN20) at individual levels over a two-week period and used anchor-based and 10-point MIDs to estimate if change is clinically meaningful; thereafter, we explored the direction and magnitude of changes.

**Results:**

Between 8.8% and 66.3% of the patients had actual changes in estimated scales. While 16.3%-60.9% and 8.8%-59.8% of the patients changed to a clinically relevant extent using anchor-based and 10-point MIDs in any scale, respectively. Changes were mostly in the functional than symptom scales and mostly minor, i.e., changes between ‘not at all’ and ‘a little’ or ‘a little’ and ‘quite a bit.’

**Conclusion:**

10-point compared to anchor-based MIDs underestimates clinically relevant changes. Therefore, the application of different MIDs to the same research question can lead to diverse result interpretations. As most changes were minor, it could be argued if these reflect actual relevant changes for an individual or that the current response scale lacks sufficient differentiating ability, warranting further research over the best method to evaluate individual-level changes.

**Supplementary Information:**

The online version contains supplementary material available at 10.1007/s11136-025-04029-3.

## Introduction

Glioma is the most common primary malignant brain tumor in adults. Both the presence of a tumor and its treatment can induce changes in different health-related quality of life (HRQoL) domains, including functional impairments (physical, social, role, and neurocognitive) and symptom burden (fatigue, pain, difficulty communicating, motor deficits, seizures, and so on) in patients with glioma. Despite multimodal treatment, the prognosis of most patients with glioma remains poor and varies considerably depending on the tumor’s molecular-genetic profiles. Patients with astrocytoma isocitrate dehydrogenase (IDH) mutant have a median survival of 1–11 years compared to 8–18 years for oligodendroglioma, IDH mutant, 1p/19q codeleted [16],van den [21]. On the contrary, patients with glioblastoma, IDH wild type, the most common and severe subtype, have a median survival of 9–21 months [16],van den [21]. Given the incurable nature of the disease and limited survival time, it is of utmost importance to maintain the patient’s HRQoL throughout the disease course as much as possible. This can be achieved by timely initiation of antitumor treatment and symptomatic treatment but also requires accurate interpretation of the patient’s change in HRQoL scores over time, which will help clinicians in making informed decisions about possible future interventions.

The most commonly used questionnaires to assess HRQoL outcomes in studies with brain tumor patients are the European Organization for Research and Treatment of Cancer (EORTC) quality-of-life questionnaire (QLQ)-C30 [1] and its brain-tumor-specific module, the EORTC QLQ-BN20 [7, 19]. Participants respond to the items in these questionnaires on 4-point Likert scales ranging from ‘not at all’ to ‘very much,’ except for the global health (GH) status/QoL scale, using a 7-point Likert scale. Several studies have shown that patients with glioma experience changes in HRQoL scores over time during the disease course and treatment [4, 5, 15]. However, not all HRQoL changes could be considered clinically meaningful for patients. To determine if a change in HRQoL is meaningful, minimally important differences (MIDs) have been established [10, 14]. MIDs is the smallest change in HRQoL scores that patients perceive as meaningful and that would justify a change in management [6, 7, 13]. For many years, clinically relevant changes in the EORTC scale scores were set at 10 points [2, 3, 14]. Recently, it has been suggested that these 10-point MIDs are too simplistic, influence the interpretation of outcomes [6, 7, 13], and do not take the direction of change (improvement or deterioration) into account [2, 3]. In 2021, anchor-based MIDs for group-level change in the EORTC QLQ-C30 scales were developed specifically for patients with glioma, and these ranged between 4 and 11 points, showing that the 10-point cutoff can underestimate clinically relevant differences [6, 7]. Notably, the MIDs for EORTC questionnaires are developed for the interpretation of between- or within-group-level differences only and not for individual-level change. Group-level change reflects average shifts in HRQoL scores across a study population, whereas individual-level change accounts for changes in HRQoL scores experienced by each patient. Clinically relevant group-level changes are typically calculated as mean differences in HRQoL scores over time while comparing them to a predefined threshold for clinical relevance. Clinically relevant individual-level changes are determined by computing each patient's score change over time and calculating the percentage of patients whose HRQoL changed (deteriorated, improved) or remained stable (no change occurred) over time on a certain item or scale based on the predefined threshold for clinical relevance. Given that there are no MIDs for individual-level change, studies apply the MIDs estimated from group-level change to individual-level change in order to define changes that are clinically meaningful. For instance, several studies reported on the percentage of patients whose HRQoL changed to a clinically meaningful extent in a specific time period [5, 22]. Currently, it is unclear what the consequences of applying different group-level MIDs to the individual-level change are in terms of interpretation of results. This is relevant because the EORTC questionnaires comprise multi-item and single-item scales, such as in the EORTC QLQ-C30 [1] and its brain-tumor-specific module, the EORTC QLQ-BN20 [7, 19]. Particularly single-item scales of the EORTC questionnaires may not have sufficient granularity to measure changes of such magnitude [6, 7, 11]. In addition, it is unknown whether patients can differentiate between the different levels of severity (e.g., a little versus quite a bit of headache), particularly over time. Furthermore, the majority of studies on brain tumor populations focus on survival outcomes, tumor response, and treatment efficacy; studies on important outcomes such as PRO measures and the use of MIDs are important and increasingly gaining interest. However, accurate information on clinically relevant change scores over time in a patient will help clinicians in making well-informed decisions about possible future interventions.

The aim of our study was to examine the challenges in interpreting individual-level changes in HRQoL in patients with glioma. Therefore, we addressed the following research questions. (1) What is the impact of applying different group-level MIDs to individual-level change? (2) In which direction and to what magnitude do HRQoL scores change? (3) Do changes in HRQoL relate to the response format of the items (4-point Likert scale) and type (functional scales versus symptom scales) of the HRQoL outcomes? Ultimately, this study may guide how to best assess and interpret clinically relevant changes in HRQoL outcomes over time at the individual level as well as foster the development of anchor- or distribution-based MIDs for individual-level changes.

## Methods

### Study design and patient population

A longitudinal study was conducted using data from glioma patients included in a previously conducted randomized prospective study (RPS) [15]. This previously conducted RPS comprised patients aged 18 years and older recruited at Haaglanden Medical Center in The Hague, The Netherlands, between July 2016 and July 2018, who had radiologically suspected glioma or histologically confirmed grade 2–4 glioma based on the World Health Organization (WHO) 2016 classification criteria [12]. Patients were randomized into two groups (1:1 ratio), where the timing of the second measurement differed: both groups completed HRQoL questionnaires at baseline t0 (i.e., the day of the magnetic resonance imaging (MRI) scan). Group 1 completed their second HRQoL assessment immediately before consultation with the physician, while Group 2 completed their second HRQoL assessment immediately after consultation with the physician. The mean time in the RPS between t0 and follow-up HRQoL assessments (t1) was 7 days with a standard deviation of 5. From the results of the RPS, there was no significant difference in the percentage of patients that improved or deteriorated up to a clinically relevant level between groups 1 and 2. Therefore, given no significant differences in HRQoL outcomes were observed between the two groups, all patients in the study were grouped together for the current analysis. More other information about the RPS was published previously [15]. All participants gave written informed consent to participate. The institutional review board of Leiden University Medical Center gave approval for this study.

### Inclusion criteria

The inclusion criteria for the current study mirrored those of the previously conducted RPS, including patients at least 18 years old with radiologically suspected glioma or histologically confirmed grade 2–4 glioma according to the WHO 2016 classification criteria. From the previously conducted RPS, we also selected patients who had t0 and t1 HRQoL assessments for inclusion in the current study. Nevertheless, to ensure a homogenous population in terms of determining changes over time, we only selected 92/100 (92%) patients whose time between the completion of the two HRQoL assessment forms (t0 and t1) ranged between 1 and 14 days. Moreover, the 1–14 day follow-up period was a well-thought-out period and was chosen because within the period of 14 days, according to psychometric papers, no clinically relevant change is expected to occur if the population is relatively in a stable state. Therefore, it enables us to assess the challenges in the application of diverse MIDs to an individual-level change as well as the challenges in the use of the 4-point Likert scale.

### Instruments

HRQoL was measured with the EORTC QLQ-C30 version 3.0 [1] and the EORTC brain cancer-specific QLQ-BN20 [19]. The EORTC QLQ-C30 is a standardized 30-item questionnaire developed to assess HRQoL of cancer patients participating in clinical trials and routine care. The questionnaire consists of functioning scales such as physical functioning (PF), role functioning (RF), emotional functioning (EF), cognitive functioning (CF), and social functioning (SF); single-item symptom scales such as dyspnea (DY), appetite loss (AP), insomnia (SL), constipation (CO), diarrhea (DI), and financial difficulties (FI); multi-item symptom scales such as fatigue (FA), pain (PA), and nausea and vomiting (NV); and a GH status/QoL score. The EORTC QLQ-BN20 is a brain tumor-specific 20-item questionnaire containing multi-item symptom scales including future uncertainty (FU), visual disorder (VD), motor dysfunction (MD), and communication deficit (CD); and seven single-item symptom scales including headaches (HA), seizures (SE), drowsiness (DR), hair loss (HL), itchy skin (IS), weakness of legs (WL), and bladder control (BC). All items in both questionnaires were scored on a 4-point Likert scale ranging from ‘not at all’ to ‘very much,’ except for the GH status/QoL scale, where a 7-point Likert scale was used. Following the EORTC Scoring Manual [8], raw scores were linearly transformed to a score between 0 and 100. A higher score reflects better functioning on the functional and GH status/QoL scales, while a higher score reflects increased symptom burden on the symptom scales. A decline in functioning score or increase in symptom score over a period indicates deterioration; an increase in functioning score or decline in symptom score over time indicates improvements; no (clinically meaningful) change in score between two time points indicates a stable state over time. However, not all changes over time in HRQoL scales are thought to be clinically relevant for patients. Therefore, to determine if a change in HRQoL score is clinically relevant, different MIDs are available: a general 10-point MID developed for between- or within-group-level differences is applicable to both the EORTC QLQ-C30 and QLQ-BN20, [14] and anchor-based MIDs developed specifically for patients with glioma to detect between- and within-group changes for the EORTC QLQ-C30 scales only [6, 7]. The anchor-based MIDs for interpreting within-group differences ranged between 4 and 12 points for improvement and between − 4 and − 14 points for deterioration. The anchor-based MIDs for the between-group difference ranged between 4 and 9 for improvement and between − 4 and − 16 for deterioration [6, 7].

### Clinical and sociodemographic variables

We collected clinical and sociodemographic variables, such as age, sex, time since diagnosis, time between T0 and T1, Karnofsky Performance Status (KPS), tumor type, radiological response on MRI, hemisphere, surgical resection, tumor treatments, marital status, use of dexamethasone, antiseizure medication, and education level***.***

## Statistical analysis

### Descriptive statistics

Descriptive statistics were used to describe sociodemographic, clinical, and HRQoL variables. Depending on the type and distribution of the variables, means with their standard deviations, medians with their interquartile ranges (IQR), or numbers and percentages were reported.

### The impact of applying diverse MIDs in change interpretation

Firstly, using linearly transformed scores, we calculated the percentage of individuals that remained stable and those with an actual change (a change without the application of the MIDs) over a two-week period. Thereafter, 10-point MID and anchor-based MIDs developed for between- or within-group-level differences were used to determine changes that are clinically meaningful over the course of the two-week period. The 10-point MID was applied to both the EORTC QLQ-C30 and QLQ-BN20 scales, while the anchor-based MIDs were only available for the EORTC QLQ-C30 scales [6, 7].

### The direction and magnitude of changes using the response format

Using the 4-Likert scale, we examined the direction of change (i.e., the percentage of patients that improved or deteriorated or remained stable) in each item and scale by dichotomizing each single item into ‘no issues’ (Likert score of 1) versus ‘issues’ (Likert scores of 2–4), and subsequently calculating the percentages of patients that moved from not having an issue to having an issue, and vice versa, in the two-week period. Next, for a multi-item scale, a patient was defined as ‘changed’ when one of the items in that specific scale changed over time from ‘no issue’ to ‘issue’ or vice versa. A sensitivity analysis was performed using another definition of change: if at least 50% of the items in a multi-item scale changed from ‘no issue’ to ‘issue’ (or vice versa), the patient was classified as ‘changed’. For example, on a three-item scale, a patient was classified as ‘changed’ when two or three of the items in the scale changed.

Subsequently, we investigated the magnitude of the change (for example, the change between “not at all” and “a little” or “not at all” and “very much”) over the two-week period by calculating the proportion of patients that showed minor changes, defined as a change by one point (i.e., between “not at all” and "a little," between "a little" and "quite a bit," or between "quite a bit" and “very much”). In addition, the percentage of patients showing moderate and major changes, defined as a change by two or three points (between “not at all” and “quite a bit" or between "a little" and "very much", or between “not at all” and “very much”, respectively), was calculated.

In the analyses using the 4-Likert scale in estimating the direction and magnitude of the change, GH was excluded because it has a different numbering of the response scale ranging from 1 to 7 points, which is different from other items and scales with a 4-response scale.

Finally, we assessed at an individual level if patients changed more in symptoms than in the functional scales. We did this by calculating the mean score of all the symptom scales at t0 and also at t1. Then, the mean score difference between t0 and t1 was assessed while a clinically relevant change was evaluated using a 10-point MID. Thereafter, the percentage of patients that remained stable or changed (deteriorated or improved) was calculated. This same process was repeated for all the functioning scales combined together. All the functioning scales were grouped together as well as all symptom scales specifically in order to get insight into if patients have difficulty quantifying change in symptom scales compared to the functioning scale. In this final analysis, the scales of FI and FU were removed because they represent the economic and future unpredictability impacts of the tumor and its treatment rather than symptoms or functional impairments arising from the tumor and its treatment. Additionally, GH was excluded because it evaluates patients’ overall health status and quality of life rather than specific HRQoL domains.

## Results

### Study and patient characteristics

Out of the 100 patients in the RPS, 92 patients whose time between the completion of HRQoL forms was between 1 and 14 days were eligible for these current analyses. Of these 92 patients, 44% (40/92) were diagnosed with glioblastoma, and 57% (52/92) were male. The median age at tumor diagnosis was 56 (IQR: 14) years; the median time since tumor diagnosis was 3 (IQR: 6) months; and the median time between the first and subsequent HRQoL assessments was 5 (IQR: 3) days. The median KPS score was 90 (IQR: 10). Most patients, 87% (80/92), had stable disease, with 47/92 (51%) not receiving active antitumor treatment (Table 1).

**Table 1:** Sociodemographic and clinical characteristics of the study population

Patient characteristics	Statistics
Total number of patients	**92**
Age; median (IQR) years	56 (14)
Sex; n (%)	
Male	52 (57)
Female	40 (43)
Time since diagnosis; median (IQR) months	3 (6)
Time between T0 and T1; median (IQR) days	5 (3)
KPS; median (IQR)	90 (10)
Tumor type; n (%)	
Glioblastoma	40 (43%)
Other gliomas*	52 (57%)
Radiological response on MRI; n (%)	
Minor disease response	3 (3%)
Stable disease response	80 (87%)
Progressive disease response	9 (10%)
Hemisphere; n (%)	
Left	43 (47%)
Right	47 (51%)
Both	2 (2%)
Surgery n (%)	
Biopsy only	12 (13%)
Partial resection	7 (7.6%)
Total resection	72 (78.3%)
Unknown	1 (1%)
Prior antitumor treatment n (%)	
Surgery only	2 (2%)
Surgery and chemotherapy	19 (21%)
Surgery and radiotherapy	6 (7%)
Surgery, chemo and radiotherapy	64 (70%)
Unknown	1 (1%)
Current antitumor treatment; n (%)	
No active treatment	47 (51%)
Chemotherapy	40 (43%)
Others	5 (5%)
Marital status; n (%)^#^	
With partner	75 (82%)
Without partner	17 (18%)
Dexamethasone; n (%)	
Yes	10 (11%)
No	82 (89%)
Antiseizure medication; n (%)	
Yes	51 (55%)
No	41 (45%)
Level of education; n (%)^$^	
Lower	51 (55%)
Higher	41 (45%)

IQR: Interquartile range, T0: first measurement moment at the day of the MRI, T1: second measurement moment before or after the consultation with the physician, KPS: Karnofsky Performance Status, n: number

*others: astrocytoma, oligodendroglioma, oligoastrocytoma, anaplastic astrocytoma, oligoastrocytoma and oligodendroglioma, glioblastoma, and ependymoma who were in either stable or progressive stage at MRI

#A partner can be a married, cohabiting, or non-cohabiting partner, while a non-partner can be single, divorced, or widowed.

^$^Level of education: Lower educational status includes primary school, lower secondary school, upper secondary school, and post-secondary, non-tertiary school; higher level of education includes short-cycle tertiary, bachelor or equivalent, master or equivalent, and doctoral or equivalent

### The impact of applying diverse MIDs in change interpretation

**Fig. 1:**
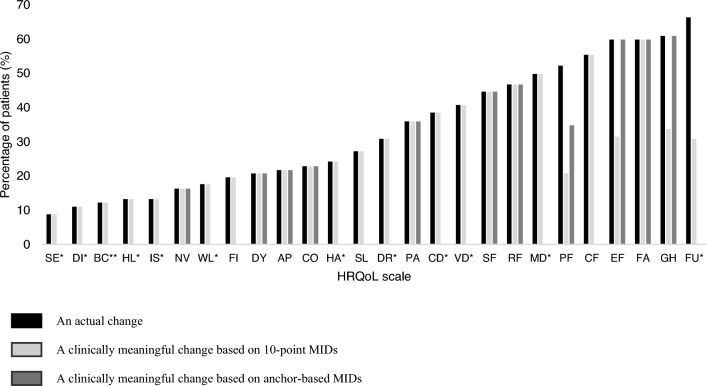
Percentage of patients in the study (n = 92) with an actual change score, a change score using 10-point MID, and a change score using anchor-based MID**.** Health related quality of life: HRQoL, Global health status: GH, Physical functioning: PF, Role functioning: RF, Emotional functioning: EF, Cognitive functioning: CF, Social functioning: SF, Fatigue: FA, Nausea and vomiting: NV, Pain: PA, Dyspnea: DY, Sleep: SL, Appetite loss: AP, Constipation: CO, Diarrhea: DI, Financial difficulties: FI, Future uncertainty: FU, Visual deficits: VD, Motor dysfunction: MD, Communication deficit: CD, Headache: HA, Seizures: SE, Drowsiness: DR, Hair loss: HL, Itchy skin: IS, Weakness of the leg: WL, Bladder control: BC. Available case analysis was performed. Available case analysis was performed with *total number of patients = 91, ** total number of patients = 90. Anchor-based MIDs were only available for certain EORTC QLQ-C30 scales

Results from linearly transformed scores showed that the percentage of patients that had an actual change in HRQoL score was similar compared to change estimated using either 10-point MIDs or anchor-based MIDs, except in certain scales. For instance, differences in the percentage of patients that had actual change compared to clinically relevant change were only present in PF (actual 52% vs. anchor-based 35% vs. 10-point 21%), EF (actual 60% vs. anchor-based 60% vs. 10-point 32%), GH (actual 61% vs. anchor-based 61% vs. 10-point 34%), and FU (actual 66% vs. 10-point 31%). For the changes that were clinically relevant within the 2 weeks, the 10-point MID showed that the percentage of patients with a clinically relevant change ranged between 9% (improved 4% and deteriorated 4%) for SE and 60% (improved 44% and deteriorated 16%) for FA. Similar results were found when the anchor-based MIDs were used, except for three scales, where the anchor-based MIDs resulted in a higher percentage of patients that could be classified as changed; for instance, GH: 34% using 10-point MID (improved 23% and deteriorated 11%) versus 61% using anchor-based MIDs (improved 21% and deteriorated 40%); PF: 21% using 10-point MID (improved 10% and deteriorated 11%) versus 35% using anchor-based MIDs (improved 24% and deteriorated 11%); and EF: 32% using 10-point MID (improved 15% and deteriorated 16%) versus 60% using anchor-based MIDs (improved 32% and deteriorated 28%). Furthermore, most changes occurred in the multi-item scales, with a range of 60% (improved 44% and deteriorated 16%) and 16.3% (improved 12% and deteriorated 4%) of the patients changing in FA and NV, compared to the single items, where a range of 30.8% (improved 20% and deteriorated 11%) and 8% (improved 4% and deteriorated 4%) of the patients changed in DR and SE, respectively (Fig. 1, Table 2, and Supplementary Table 2).

**Table 2:** Percentage of patients with a change in HRQoL score using four types of definitions

Type of change	Change in HRQoL scores estimated using linearly transformed scores												Actual change in HRQoL scores estimated from the recategorized 4-Likert scale			
Type of change	An actual change in HRQoL scores was estimated without using MIDs, n (%)				A clinically meaningful change in the HRQoL was defined based on the MID of 10-point difference, n (%)				A clinically meaningful change in the HRQoL was defined based on the anchor-based MID, n (%)				An actual change in HRQoL was defined if any of the items in a scale changed, n (%)			
HRQoL scales/direction of change	Stable	Changed	Direction of change		Stable	Changed	Direction of change		Stable	Changed	Direction of change		Stable	Changed	Direction of change	
			Improved	Deteriorated			Improved	Deteriorated			Improved	Deteriorated			Improved	Deteriorated
PF	44 (47.8)	48 (52.2)	22 (23.9)	26 (28.2)	73 (79.3)	19 (20.7)	9 (9.8)	10 (10.9)	60(65.2)	32(34.8)	22 (23.9)	10 (10.9)	62 (67.4)	30 (32.6)	15 (16.3)	15 (16.3)
RF	49 (53.3)	43 (46.7)	14 (15.2)	29 (31.5)	49 (53.3)	43 (46.7)	14 (15.2)	29 (31.5)	49(53.3)	43(46.7)	14 (15.2)	29 (31.5)	69 (75.0)	23 (25.0)	14 (15.2)	9 (9.8)
EF	37 (40.2)	55 (59.8)	29 (31.5)	26 (28.3)	63 (68.5)	29 (31.5)	14 (15.2)	15 (16.3)	37 (40.2)	55(59.8)	29 (31.5)	26 (28.2)	50 (54.6)	42 (45.7)	17 (18.5)	25 (27.2)
CF	41 (44.6)	51 (55.4)	20 (21.7)	31 (33.7)	41 (44.6)	51 (55.4)	20 (21.7)	31 (33.7)	-	-	-	-	62 (67.8)	30 (32.2)	20 (21.7)	10 (10.9)
SF	51(55.4)	41 (44.6)	9(9.8)	32(34.8)	51(55.4)	41 (44.6)	9(9.8)	32(34.8)	51 (55.4)	41 (44.6)	9 (9.8)	32 (34.8)	63 (68.5)	29 (31.5)	22 (23.9)	7 (7.6)
GH	39 (39.1)	56 (60.9)	19 (20.7)	27 (40.2)	61 (66.3)	41(33.7)	31(22.8)	10 (10.9)	36(39.1)	56(60.9)	19 (20.7)	37 (40.2)	NA	NA	NA	NA
FA	37 (40.2)	55 (59.8)	40 (43.5)	15 (16.3)	37 (40.2)	55 (59.8)	40 (43.5)	15 (16.3)	37(40.2)	55(59.8)	40 (43.5)	15 (16.3)	61 (66.3)	31(33.7)	20 (21.7)	11 (12.0)
NV	77 (83.7)	15 (16.3)	11 (12.0)	4 (4.3)	77 (83.7)	15 (16.3)	11 (12.0)	4 (4.3)	77(83.7)	15(16.3)	11 (12.0)	4 (4.3)	79 (85.9)	13 (14.1)	9 (9.8)	4 (4.3)
PA	59 (64.1)	33 (35.9)	15 (16.3)	18 (19.6)	59 (64.1)	33 (35.9)	15 (16.3)	18 (19.6)	59(64.1)	33(35.9)	15 (16.3)	18 (19.6)	66 (71.7)	26 (28.2)	13 (14.1)	13 (14.1)
DY	73 (79.3)	19 (20.7)	13 (14.1)	6 (6.5)	73 (79.3)	19 (20.7)	13 (14.1)	6 (6.5)	73(79.3)	19(20.7)	13 (14.1)	6 (6.5)	80 (87.0)	12 (13.0)	7 (7.6)	5 (5.4)
SL	67 (72.8)	25 (27.2)	14 (15.2)	11 (12.0)	67 (72.8)	25 (27.2)	14 (15.2)	11 (12.0)	-	-	-	-	79 (85.9)	13 (14.1)	7 (7.6)	6 (6.5)
AP	72 (78.3)	20 (21.7)	15 (16.3)	5 (5.4)	72 (78.3)	20 (21.7)	15 (16.3)	5 (5.4)	72(78.3)	20(21.7)	15 (16.3)	5 (5.4)	80 (87.0)	12 (13.0)	9 (9.8)	3 (3.2)
CO	71 (77.2)	21 (22.8)	11 (12.0)	10 (10.9)	71 (77.2)	21 (22.8)	11 (12.0)	10 (10.9)	71(77.2)	21(22.8)	11 (12.0)	10 (10.9)	80 (87.0)	12 (13.0)	6 (6.5)	6 (6.5)
DI*	81 (89.0)	10 (11.0)	4 (4.4)	6 (6.6)	81 (89.0)	10 (11.0)	4 (4.4)	6 (6.6)	-	-	-	-	82 (90.1)	9 (9.9)	4 (4.4)	5 (5.5)
FI	74 (80.4)	18 (19.6)	11 (12.0)	7 (7.6)	74 (80.4)	18 (19.6)	11 (12.0)	7 (7.6)	-	-	-	-	78 (84.8)	14 (15.2)	7 (7.6)	7 (7.6)
FU*	69 (75.8)	61 (66.3)	40 (43.5)	21 (22.8)	63 (69.2)	28 (30.8)	19 (20.9)	9 (9.9)	-	-	-	-	44 (48.4)	47 (51.6)	30 (33.0)	17 (18.7)
HA*	54 (59.3)	22 (24.2)	14 (15.4)	8 (8.8)	69 (75.8)	22 (24.2)	14 (15.4)	8 (8.8)	-	-	-	-	74 (81.3)	17 (18.7)	10 (11.0)	7 (7.7)
VD*	46 (50.5)	37 (40.2)	22 (24.2)	15 (16.5)	54 (59.3)	37 (40.7)	22 (24.2)	15 (16.5)	-	-	-	-	58 (63.7)	33 (36.3)	18 (19.8)	15 (16.5)
MD*	56 (61.5)	45 (49.8)	21 (23.1)	24 (26.4)	46 (50.5)	45 (49.8)	21 (23.1)	24 (26.4)	-	-	-	-	51 (56.0)	40 (44.0)	18 (19.8)	22 (24.2)
CD*	83 (91.2)	35 (38.5)	21 (23.1)	14 (15.4)	56 (61.5)	35 (38.5)	21 (23.1)	14 (15.4)	-	-	-	-	63 (69.2)	28 (30.8)	12 (13.2)	16 (17.6)
SE*	63 (69.2)	8 (8.8)	4 (4.4)	4 (4.4)	83 (91.2)	8 (8.8)	4 (4.4)	4 (4.4)	-	-	-	-	84 (92.3)	7 (7.7)	3 (3.3)	4 (4.4)
DR*	79 (86.8)	28 (30.8)	18 (19.8)	10 (11.0)	63 (69.2)	28 (30.8)	18 (19.8)	10 (11.0)	-	-	-	-	73 (80.2)	18 (19.8)	11 (12.1)	7 (7.7)
HL*	79 (86.8)	12 (13.2)	6 (6.6)	6 (6.6)	79 (86.8)	12 (13.2)	6 (6.6)	6 (6.6)	-	-	-	-	84 (92.3)	7 (7.7)	3 (3.3)	4 (4.4)
IS*	75 (82.4)	12 (13.2)	5 (5.5)	7 (7.7)	79 (86.8)	12 (13.2)	5 (5.5)	7 (7.7)	-	-	-	-	83 (91.2)	8 (8.8)	2 (2.2)	6 (6.6)
WL*	79 (87.8)	16 (17.6)	6 (6.6)	10 (11.0)	75 (82.4)	16 (17.6)	6 (6.6)	10 (11.0)	-	-	-	-	78 (85.7)	13 (14.3)	6 (6.6)	7 (7.7)
BC**	69 (75.8)	11 (12.2)	9 (9)	2 (2.2)	79 (87.8)	11 (12.2)	9 (9)	2 (2.2)	-	-	-	-	82 (91.1)	8 (8.9)	6 (6.7)	2 (2.2)

HRQoL: Health-related quality of life, Physical functioning: PF, Role functioning: RF, Emotional functioning: EF, Cognitive functioning: CF, Social functioning: SF, Fatigue: FA, Nausea and vomiting: NV, Pain: PA, Dyspnea: DY, Sleep: SL, Appetite loss: AP, Constipation: CO, Diarrhea: DI, Financial difficulties: FI, Future uncertainty: FU, Visual deficits: VD, Motor dysfunction: MD, Communication deficit: CD, Headache: HA, Seizures: SE, Drowsiness: DR, Hair loss: HL, Itchy skin: IS, Weakness of the leg: WL, Bladder control: BC. NA, not applicable—due to the exclusion of global health status, which is measured using a seven-point Likert scale ranging from 1 to 7. Categorizing these scores as binary may introduce bias, since we are not sure what each score means. E.g., a 1 in a 4-point Likert score means “not at all,” while 4 means “very much.” MID: Minimal important difference **(—) Dashed lines: ** there were no estimated anchor-based MIDs for the estimation of clinically relevant change

Available case analysis was performed with * a total number of patients = 91 and ** total number of patients = 90

Results on actual change using the recategorized 4-Likert scales showed that a large proportion of patients could be classified as changed, with the least actual change seen in HL and SE (8%, improved 3% and deteriorated 4.5%), while the most changes occurred in FU (52%, improved 33% and deteriorated 19%), EF (46%, improved 19% and deteriorated 27%), and MD (44%, improved 20% and deteriorated 24%), etc. (Fig. 1 and Table 2).

### The direction and magnitude of change using the response format

Results on the actual change using the recategorized 4-Likert scales showed that patients changed within a 2-week period, and these changes ranged between 2% in NV item 2 and 25% in either FU item 2 or EF item 3 (supplementary Table 1). The direction of these changes (improved and deteriorated) varied between items and scales over time. For the item-level change, the percentage of patients that improved ranged between 1% in PF items 2 and 3 and 17% in FU item 2. The percentage of patients that deteriorated was between 1% in VD item 1 and 17% in EF item 1 (Supplementary Table 1). For the scale-level change (main analysis: change defined when any of the items in the scale had changed), the percentage of patients that improved ranged between 2% in IS and 33% in FU. In contrast, the percentage of patients that deteriorated ranged between 2% in BC and 27% in EF (Table 2). The sensitivity analyses, in which we used a more flexible definition—that is, patients were classified as changed where at least 50% of the items could be categorized as changed—showed that the majority of the patients remained stable, with no change (0%) in PF and only a 1% change observed in NV and FU, while the largest change occurred in DR (20%), followed by HA (19%) (Supplementary Table 2).

Table 3 shows the diversity in the magnitude of change within a short period of 2 weeks. Most changes were minor (i.e., a change at one point between “not at all” and "a little," between "a little" and "quite a bit," or between "quite a bit" and “very much”). For minor changes, the percentages of patients that changed ranged between 66.7% (in EORTC QLQ-BN20 item number 16) and 100% (in EORTC QLQ-C30 item numbers 2, 3, 9, 14, 15, 18, 22, 24, and EORTC QLQ-BN20 item numbers 1, 6, and 7); see Table 3. Notably, in 96%-100% of all the items, the order in minor change was from “a little to not at all” and “not at all to a little.” In addition, in 83%-90% of all the items estimated, the order was from "a little to quite a bit" and “quite a bit to a little.” And finally, in 35%-56% of all the items estimated, minor changes were from “quite a bit to very much” and “very much to quite a bit,” respectively (Supplementary Table 3). Furthermore, moderate change (change at two points, between “not at all” and “quite a bit" or between "a little" and "very much") occurred at 39 (78%) of the items, while major changes (change at three points, between “not at all” and “very much”) occurred in only 4 (8%) of all the estimated items, namely FI, MD, DR, and HL (Table 3).

**Table 3:** Magnitude of change, showing the percentage of patients that remained stable or changed by one, two, and three points

EORTC QLQ-C30	Item numbers	Magnitude of change, Number (%) of patients that changed		
		Minor change: at one point^#^	Moderate change: at two points ^#^	Major change: at three points^#^
Physical functioning	1	21 (87.5)	3 (12.5)	0 (0.0)
	2	20 (100)	0 (0.0)	0 (0.0)
	3	17 (100)	0 (0.0)	0 (0.0)
	4	18 (85.7)	3 (14.3)	0 (0.0)
	5	6 (85.7)	1 (14.3)	0 (0.0)
Emotional functioning	21	29 (87.9)	4 (12.1)	0 (0.0)
	22	32 (100.0)	0 (0.0)	0 (0.0)
	23	34 (94.4)	2 (5.6)	0 (0.0)
	24	30 (100.0)	0 (0.0)	0 (0.0)
Role functioning	6	32 (88.9)	4 (11.1)	0 (0.0)
	7*	26 (92.9)	2 (7.1)	0 (0.0)
Cognitive functioning	20	27 (81.8)	6 (18.2)	0 (0.0)
	25	29 (87.9)	4 (12.1)	0 (0.0)
Social functioning	26	22 (91.7)	2 (8.3)	0 (0.0)
	27	28 (80.0)	7 (20.0)	0 (0.0)
Fatigue	10	31 (91.2)	3 (8.8)	0 (0.0)
	12	32 (97.0)	1 (3.0)	0 (0.0)
	18*	28 (100.0)	0 (0.0)	0 (0.0)
Nausea and vomiting	14	13 (100.0)	0 (0.0)	0 (0.0)
	15	3 (100.0)	0 (0.0)	0 (0.0)
Pain	9	24 (100.0)	0 (0.0)	0 (0.0)
	19	20 (83.3)	4 (16.7)	0 (0.0)
Dyspnea	8	18 (94.7)	1 (5.3)	0 (0.0)
Sleep	11	24 (96.0)	1 (4.0)	0 (0.0)
Appetite loss	13	18 (90.0)	2 (10.0)	0 (0.0)
Constipation	16	19 (90.5)	2 (9.2)	0 (0.0)
Diarrhea	17	7 (70.0)	3 (30.0)	0 (0.0)
Financial difficulties	28	16 (88.9)	1 (5.6)	1 (5.6)
EORTC QLQ-BN20				
Future uncertainty	1*	24 (100.0)	0 (0.0)	0 (0.0)
	2*	27 (90.0)	3 (10.0)	0 (0.0)
	3*	26 (96.3)	1 (3.7)	0 (0.0)
	5*	27 (96.4)	1 (3.6)	0 (0.0)
Headache	4	17 (77.3)	5 (5.4)	0 (0.0)
Visual deficits	6**	6 (100.0)	0 (0.0)	0 (0.0)
	7*	22 (100.0)	0 (0.0)	0 (0.0)
	8*	24 (92.3)	2 (7.7)	0 (0.0)
Seizures	9*	24 (92.3)	2 (7.7)	0 (0.0)
Motor dysfunction	10*	6 (75.0)	2 (25.0)	0 (0.0)
	15*	24 (77.4)	5 (16.1)	2 (6.5)
	19*	24 (96.0)	1 (4.0)	0 (0.0)
Communication deficit	11*	19 (86.4)	3 (13.6)	0 (0.0)
	12*	11 (73.3)	4 (4.3)	0 (0.0)
	13*	25 (89.3)	3 (10.7)	0 (0.0)
Drowsiness	14*	26 (92.9)	1 (3.6)	1 (3.6)
Hair loss	16*	8 (66.7)	3 (25.0)	1 (8.3)
Itchy skin	17*	11 (91.7)	1 (8.3)	0 (0.0)
Weakness of the leg	18*	14 (87.5)	2 (12.5)	0 (0.0)
Bladder control	20**	9 (81.8)	2 (2.2)	0 (0.0)

A total of 92 participants was included in the analysis. In case of missing data, an available case analysis was performed with a *total number of patients = 91 and ** total number of patients = 90. **#** One point: change between "not at all" and "a little," "a little" and "quite a bit,” “quite a bit” and “very much.”** ##** Two points: change between “not at all” and “quite a bit,” “a little” and “very much.”** ###** Three points: “not at all” and “very much

### Relation between changes in HRQoL scores and the type of scale

Lastly, we investigated whether the changes in HRQoL scores (based on the 10-point MID) were related to the type of scales, i.e., functional scale or symptom scale. As shown in Table 4, at an individual level, more patients with clinically relevant changes (29%) and fewer patients with stable status (71%) were observed in the functional scales as compared to the symptom scales (changed: 11% and stable: 89%).

**Table 4:** Percentage of patients with a clinically relevant change (based on the 10-point MID) in the functional and symptom scales separately

	Functional scales * n (%) of changed based on 10-point MID	Symptom scales* n (%) of changed based on 10-point MID
Stable	65 (70.7)	82 (89.1)
Changed Deteriorated Improved	27 (29.3) 19 (20.7) 8 (8.7)	10 (10.9) 4 (4.3) 6 (6.5)

n (%): number with percentages; MID: Minimally important different

*The scales of financial difficulty, GH, and future uncertainty (FU) were removed from this analysis because we considered these neither symptoms nor functional scales

## Discussion

The result of our study showed that the interpretation of the individual-level change in the EORTC QLQ-C30 scores in patients with glioma relies on the definition that is used to evaluate a clinically relevant change. Indeed, the different MIDs (the 10-point MID and anchor-based MIDs) resulted in different interpretations. In addition, we found that the direction of change in the analysis of the 4-point Likert scale varied between items and scales over time, as the percentage of patients that improved ranged between 2% in IS and 33% in FU. The percentage of patients that deteriorated ranged between 2% in BC and 27% in EF. Moreover, the changes in HRQoL scores were mostly minor, with the percentages of patients that changed ranging between 66.7% and 100% in some scales. Also, most changes were observed in the functional scales as compared to the symptom scales.

In line with what is previously known, [2, 3] we observed that the 10-point MID, as compared to anchor-based MIDs, underestimates clinically relevant changes. This is evident, as some anchor-based MIDs have values less than 10 points. Additionally, when actual changes (arising from linearly transformed scores and change when any of the items in the response scales changed) were compared to 10-point MID, 10-point MID resulted in an underestimation of change, especially in PF, EF, FU, and GH. Nevertheless, this finding emphasizes that the application of different methods in change analysis to the same research question can lead to diverse interpretations of results on some scales, consequently altering the clinically relevant information that could be given to health policymakers, clinicians, and patients for better health management. In clinical practice, information on HRQoL outcomes is taken into account when deciding on future interventions. Therefore, a reliable cutoff to determine what is clinically relevant on the individual level is needed [9, 17].

Given that there are no (anchor-based or distribution-based) MIDs available for individual-level change for both EORTC-QLQ C30 and QLQ-BN20 questionnaires, our study applied the group-level MID to individual-level change. The application of the group-level MID to individual-level change in this study and most of the HRQoL studies has implications. Several scales of the EORTC questionnaires, particularly the single items, may lack sufficient granularity to measure changes. For instance, at the individual level, changes in single items are usually large, consequently resulting in a clinically relevant change. Substantially, it is warranted that MIDs need to be developed that can be applied in the interpretation of individual-level change. Only then can we determine whether a change in HRQoL outcome truly represents a clinically relevant change at the individual level.

We did not expect that such a large proportion of patients would experience a clinically relevant change over the short 14-day period, as based on the available MIDs, particularly when most patients (87%) have stable disease. Our analysis, where we evaluated actual change—that is, if patients changed without applying MIDs or changed from ‘no issues’ to ‘issues,’ or vice versa—also showed that patients do report changes in their health status. We did also find that patients experienced more often clinically relevant changes in the functional scales as compared to the symptom scales, suggesting that patients may find it more difficult to quantify their level of functioning over time. Moreover, in the psychometric studies, the 2-week period is deemed short enough to assume that HRQoL would not change to a clinically relevant extent. However, these findings call into question the application of a 2-week period in psychometric testing, which is often used to establish the reliability of a newly developed questionnaire. Apparently, many patients report changes, even with stable disease.

Nevertheless, most of these changes were minor. Indeed, most patients reported changes between "not at all" and “a little” or between "a little" and "quite a bit." This suggests that the response categories are semantically close, perhaps making it difficult for patients to clearly differentiate between the response options. Studies undertaken so far have shown that response scales impact patients’ answers to questionnaire items because respondents may reluctantly place themselves in a defined category, resulting in under- or over-reporting compared to an open-answer format [18, 20]. Thus, further research is needed to establish whether the current response format is most optimal.

### Strengths and limitations

One of the strengths of this study is that the compliance rate of the patients with respect to the HRQoL was optimal. Attrition bias is a serious problem in brain tumor patients in most longitudinal studies, given their poor compliance rate, partly due to death, neurocognitive problems, poor performance status, and tumor progression. Although a heterogeneous group (including astrocytoma, oligodendroglioma, oligoastrocytoma, anaplastic astrocytoma, oligoastrocytoma and oligodendroglioma, glioblastoma, and ependymoma who were in either stable or progressive condition) of patients with glioma was recruited, one of the study limitations is the convenient sampling of patients from a single center—the Haaglanden Medical Center—in which patients with a better health status participated, as reflected by their good performance status (median KPS of 90), hampering generalizability of the results to the entire population of patients with glioma. Additionally, the generalizability was also limited because of the unavailability of information on certain patient variables, including the racial and ethnic background. Considering that there are no (anchor-based or distribution-based) MIDs available for individual-level change for both EORTC-QLQ C30 and QLQ-BN20 questionnaires, the application of the group-level MID to individual-level change in this study has some implications, as it can introduce bias.

## Conclusion

In conclusion, using different analyses of change, including the application of diverse group-level MIDs, to interpret changes in HRQoL scores at the individual level may result in an under- or overestimation of relevant changes. Therefore, development of individual-level anchor- or distribution-based MIDs is needed for the interpretation of individual-level change. As most changes were minor, it could be argued if these reflect actual relevant changes or that the current response scale lacks sufficient differentiating ability, warranting further research into how to best evaluate individual-level changes.

## Supplementary Information

Below is the link to the electronic supplementary material.

Supplementary file1 (DOCX 67 KB)

## Data Availability

Data used for the current study are available upon request.
