# Supplementary material for: Challenges in interpreting individual-level changes in health-related quality of life in patients with glioma using minimally important differences (MIDs) and a 4-point Likert scale
Source: Qual Life Res. 2025 Aug 5;34(10):2809–19. doi: 10.1007/s11136-025-04029-3 (PMC12535528; doi:10.1007/s11136-025-04029-3)
Supplement: Supplementary file 1 — Supplementary file1 (DOCX 67 KB) [file 11136_2025_4029_MOESM1_ESM.docx]

**Title:**

Challenges in interpreting individual-level changes in health-related quality of life in patients with glioma using minimally important differences (MIDs) and a 4-point Likert scale.

**Authors**

Ogechukwu A. Asogwa^1^, Johan A.F. Koekkoek^1,2^, Marthe C.M. Peeters^1^, Hanneke Zwinkels^2^, Maaike J. Vos^1,2^, Linda Dirven^1,2^, Martin J.B. Taphoorn^1,2^

**Affiliations**

^1^Department of Neurology, Leiden University Medical Center, Leiden, the Netherlands

^2^Department of Neurology, Haaglanden Medical Center, the Hague, the Netherlands

**Corresponding author:**

Ogechukwu A. Edeh-Asogwa, DVM, MSc

Leiden University Medical Center

Department of Neurology

PO Box 9600, 2300 RC Leiden, the Netherlands

E-mail: [o.a.edeh-asogwa@lumc.nl](mailto:o.a.edeh-asogwa@lumc.nl)

https://orcid.org/0000-0001-6300-332X

**Supplementary Information**

**Supplementary Table 1 Percentage of patients with a change in HRQoL item score using a change defined as a change in any of the items in the scale**

| Item | Stable, n(%) | Changed, n (%) | Direction and Magnitude of change | |
| --- | --- | --- | --- | --- |
|  |  |  | Deteriorated, n (%) | Improved, n (%) |
| PF 1 | 80 (87.0) | 12 (13.0) | 3 (3.3) | 9 (9.8) |
| PF 2 | 85 (92.4) | 7 (7.6) | 6 (6.5) | 1 (1.1) |
| PF 3 | 85 (92.4) | 7 (7.6) | 6 (6.5) | 1 (1.1) |
| PF 4 | 78 (84.8) | 14 (15.2) | 6 (6.5) | 8 (8.7) |
| PF 5 | 88 (95.7) | 4 (4.3) | 2 (2.2) | 2 (2.2) |
| RF1 | 74 (80.4) | 18 (19.6) | 6 (6.5) | 12 (13.0) |
| RF2* | 79 (68.8) | 12 (13.0) | 5 (5.5) | 7 (7.7) |
| EF1 | 71 (77.2) | 21 (22.8) | 16 (17.4) | 5 (5.4) |
| EF2 | 78 (84.8) | 14 (15.2) | 7 (7.6) | 7 (7.6) |
| EF3 | 69 (75.0) | 23 (25.0) | 11 (12.0) | 12 (13.0) |
| EF4 | 78 (84.8) | 14 (15.2) | 6 (6.5) | 8 (8.7) |
| CF1 | 76 (82.6) | 16 (17.4) | 4 (4.3) | 12 (13.0) |
| CF2 | 76 (82.6) | 16 (17.4) | 7 (7.6) | 9 (9.8) |
| SF1 | 74 (80.4) | 18 (19.6) | 4 (4.3) | 14 (15.2) |
| SF2 | 72 (78.3) | 20 (21.7) | 5 (5.4) | 15 (16.3) |
| DY | 80 (87.0) | 12 (13.0) | 5 (5.4) | 7 (7.6) |
| PA1 | 74 (80.4) | 18 (19.6) | 9 (9.8) | 9 (9.8) |
| PA2 | 71 (77.2) | 21 (22.8) | 11 (12.0) | 10 (10.9) |
| FA1 | 82 (89.1) | 10 (10.9) | 3 (3.3) | 7 (7.6) |
| FA2* | 71 (77.2) | 21 (22.8) | 8 (8.7) | 13 (14.1) |
| FA3* | 78 (85.7) | 13 (14.3) | 4 (4.4) | 9 (9.9) |
| SL | 79 (85.9) | 13 (14.1) | 6 (6.5) | 7 (7.6) |
| AP | 80 (87.0) | 12 (13.0) | 3 (3.3) | 9 (9.8) |
| NV1 | 80 (87.0) | 12 (13.0) | 3 (3.3) | 9 (9.8) |
| NV2 | 90 (97.9) | 2 (2.2) | 2 (2.2) | - |
| CO | 80 (87.0) | 12 (13.0) | 6 (6.5) | 6 (6.5) |
| DI* | 82 (90.1) | 9 (9.9) | 5 (5.5) | 4 (4.4) |
| FI | 78 (84.8) | 14 (15.2) | 7 (7.6) | 7 (7.6) |
| FU1* | 80 (87.9) | 11 (12.1) | 4 (4.4) | 7 (7.7) |
| FU2* | 68 (74.7) | 23 (25.0) | 8 (8.8) | 15 (16.5) |
| FU3* | 72 (79.1) | 19 (20.9) | 6 (6.6) | 13 (14.3) |
| HA* | 74 (81.3) | 17 (18.7) | 7 (7.7) | 10 (11.0) |
| FU4* | 75 (82.4) | 16 (17.6) | 5 (5.5) | 11 (12.1) |
| VD1** | 84 (93.3) | 6 (6.7) | 1 (1.1) | 5 (5.6) |
| VD2* | 73 (80.2) | 18 (19.8) | 9 (9.9) | 9 (9.9) |
| VD3* | 71 (78.0) | 20 (22.0) | 11 (12.1) | 9 (9.9) |
| SE* | 84 (92.3) | 7 (7.7) | 4 (4.4) | 3 (3.3) |
| MD1* | 83 (91.2) | 8 (8.8) | 4 (4.4) | 4 (4.4) |
| MD2 | 70 (76.9) | 21 (23.1) | 13 (14.3) | 8 (8.8) |
| MD3 | 72 (79.1) | 19 (20.9) | 11 (12.1) | 8 (8.8) |
| CD1* | 84 (92.3) | 7 (7.7) | 4 (4.4) | 3 (3.3) |
| CD2* | 80 (87.9) | 11 (12.1) | 7 (7.7) | 4 (4.4) |
| CD3* | 73 (80.2) | 18 (19.8) | 11 (12.1) | 7 (7.7) |
| DR* | 73 (80.2) | 18 (19.8) | 7 (7.7) | 11 (12.1) |
| HL* | 84 (92.3) | 7 (7.7) | 4 (4.4) | 3 (3.3) |
| IS* | 83 (91.2) | 8 (8.8) | 6 (6.6) | 2 (2.2) |
| WL | 78 (85.7) | 13 (14.3) | 7 (7.7) | 6 (6.6) |
| BC** | 82 (91.1) | 8 (8.9) | 2(2.2) | 6 (6.7) |

*Abbreviations: HRQoL: Health-related quality of life, MID: Minimal important difference, Physical functioning: PF, Role functioning: RF, Emotional functioning: EF, Cognitive functioning: CF, Social functioning: SF, Fatigue: FA, Nausea and vomiting: NV, Pain: PA, Dyspnea: DY, Sleep: SL, Appetite loss: AP, Constipation: CO, Diarrhea*: DI, Financial difficulties: FI, Future uncertainty*: FU, Visual deficits*: VD, Motor dysfunction*: MD, Communication deficit*: CD, Headache*: HA, Seizures: SE*, Drowsiness*: DR, Hair loss*: HL, Itchy skin*: IS, Weakness of the leg*: WL, Bladder control**: BC. Available case analysis was performed with * a total number of patients=91 and ** total number of patients=90.*

**Supplementary Table 2 Percentage of patients with a change in HRQoL score using five types of definition**

| Scales | Change in HRQoL scores estimated using linearly transformed scores | | | | | | Actual change in HRQoL scores estimated from the recategorized 4-Likert scale | | | |
| --- | --- | --- | --- | --- | --- | --- | --- | --- | --- | --- |
| Scales | A clinically meaningful change in HRQoL scores was based on the 10-point MIDs, n (%) | | A clinically meaningful change in HRQOL score was based on anchor-based MIDs, n (%) | | An actual change in the HRQoL scores was estimated without using MIDs, n (%) | | Main analysis: A change in HRQoL was defined if any of the items in the scale has changed, n (%) | | Sensitivity analysis: A change in HRQoL was defined if at least 50% of the items in the scale has changed, n (%) | |
| EORTC QLQ SCALES | STABLE | CHANGE | STABLE | CHANGE | STABLE n(%) | CHANGE n(%) | STABLE | CHANGE | STABLE | CHANGE |
| *Physical functioning* | 73 (79.3) | 19 (20.7) | 60 (65.2) | 32 (34.8) | 44 (47.8) | 48 (52.2) | 62 (67.4) | 30 (32.6) | 92 (100.0) | 0 (0.0) |
| *Role functioning:* | 49 (53.3) | 43 (46.7) | 49 (53.3) | 43 (46.7) | 49 (53.3) | 43 (46.7) | 69 (75.0) | 23 (25.0) | 85 (92.4) | 7 (7.6) |
| *Emotional functioning* | 63 (68.5) | 29 (31.5) | 37 (40.2) | 55 (59.8) | 37 (40.2) | 55 (59.8) | 50 (54.6) | 42 (45.7) | 86 (93.5) | 6 (6.5) |
| *Cognitive functioning* | 41 (44.6) | 51 (55.4) | - | - | 41 (44.6) | 51 (55.4) | 62 (67.8) | 30 (32.2) | 90 (97.8) | 2 (2.2) |
| *Social functioning* | 51 (55.4) | 41 (44.6) | 51 (55.4) | 41 (44.6) | 51 (55.4) | 41 (44.6) | 63 (68.5) | 29 (31.5) | 83 (90.2) | 9 (9.8) |
| *Global health status* | 61 (66.3) | 31(33.7) | 36 (39.1) | 56 (60.9) | 36 (39.1) | 56 (60.9) | NA | NA | NA | NA |
| *Fatigue:* | 37 (40.2) | 55 (59.8) | 37 (40.2) | 55 (59.8) | 37 (40.2) | 55 (59.8) | 61 (66.3) | 31 (33.7) | 81 (88.0) | 11 (12.0) |
| *Nausea and vomiting* | 77 (83.7) | 15 (16.3) | 77 (83.7) | 15 (16.3) | 77 (83.7) | 15 (16.3) | 79 (85.9) | 13 (14.1) | 91 (98.9) | 1 (1.1) |
| *Pain* | 59 (64.1) | 33 (35.9) | 59 (64.1) | 33 (35.9) | 59 (64.1) | 33 (35.9) | 66 (71.7) | 26 (28.2) | 82 (89.1) | 10 (10.9) |
| *Dyspnea* | 73 (79.3) | 19 (20.7) | 73 (79.3) | 19 (20.7) | 73 (79.3) | 19 (20.7) | 80 (87.0) | 12 (13.0) | 80 (87.0) | 12 (13.0) |
| *Sleep* | 67 (72.8) | 25 (27.2) | - | - | 67 (72.8) | 25 (27.2) | 79 (85.9) | 13 (14.1) | 79 (85.9) | 13 (14.1) |
| *Appetite loss* | 72 (78.3) | 20 (21.7) | 72 (78.3) | 20 (21.7) | 72 (78.3) | 20 (21.7) | 80 (87.0) | 12 (13.0) | 80 (87.0) | 12 (13.0) |
| *Constipation* | 71 (77.2) | 21 (22.8) | 71 (77.2) | 21 (22.8) | 71 (77.2) | 21 (22.8) | 80 (87.0) | 12 (13.0) | 80 (87.0) | 12 (13.0) |
| *Diarrhea* * | 81 (89.0) | 10 (11.0) | - | - | 81 (89.0) | 10 (11.0) | 82 (90.1) | 9 (9.9) | 82 (90.1) | 9 (9.9) |
| *Financial difficulties* | 74 (80.4) | 18 (19.6) | - | - | 74 (80.4) | 18 (19.6) | 78 (84.8) | 14 (15.2) | 78 (84.8) | 14 (15.2) |
| *Future uncertainty* * | 63 (69.2) | 28 (30.8) | - | - | 30 (32.6) | 61 (66.3) | 44 (48.4) | 47 (51.6) | 90 (98.9) | 1 (1.1) |
| *Headache* * | 69 (75.8) | 22 (24.2) | - | - | 69 (75.8) | 22 (24.2) | 74 (81.3) | 17 (18.7) | 74 (81.4) | 17 (18.7) |
| *Visual deficits* * | 54 (59.3) | 37 (40.7) | - | - | 54 (58.7) | 37 (40.2) | 58 (63.7) | 33 (36.3) | 82 (91.1) | 8 (8.9) |
| *Motor dysfunction* * | 46 (50.5) | 45 (49.8) | - | - | 46 (50.5) | 45 (49.8) | 51 (56.0) | 40 (44.0) | 84 (92.3) | 7 (7.7) |
| *Communication deficit* * | 56 (61.5) | 35 (38.5) | - | - | 56 (61.5) | 35 (38.5) | 63 (69.2) | 28 (30.8) | 85 (93.4) | 6 (6.6) |
| *Seizures* * | 83 (91.2) | 8 (8.8) | - | - | 83 (91.2) | 8 (8.8) | 84 (92.3) | 7 (7.7) | 84 (92.3) | 7 (7.7) |
| *Drowsiness* * | 63 (69.2) | 28 (30.8) | - | - | 63 (69.2) | 28 (30.8) | 73 (80.2) | 18 (19.8) | 73 (80.2) | 18 (19.8) |
| *Hair loss* * | 79 (86.8) | 12 (13.2) | - | - | 79 (86.8) | 12 (13.2) | 84 (92.3) | 7 (7.7) | 84 (92.3) | 7 (7.7) |
| *Itchy skin* * | 79 (86.8) | 12 (13.2) | - | - | 79 (86.8) | 12 (13.2) | 83 (91.2) | 8 (8.8) | 83 (91.2) | 8 (8.8) |
| *Weakness of the leg* * | 75 (82.4) | 16 (17.6) | - | - | 75 (82.4) | 16 (17.6) | 78 (85.7) | 13 (14.3) | 78 (85.7) | 13 (14.3) |
| *Bladder control* ** | 79 (87.8) | 11 (12.2) | - | - | 79 (87.8) | 11 (12.2) | 82 (91.1) | 8 (8.9) | 82 (91.1) | 8 (8.9) |

*Abbreviations: HRQoL: Health-related quality of life, MID: Minimal important difference***.** ***-*** *there was no estimated anchor-based MIDs available for the estimation of clinically relevant change. Available case analysis was performed with *Total number of patients=91, ** total number of patients=90. NA: not applicable, due to the exclusion of global health status because it uses a 7-Likert scale ranging from 1 to 7. Categorizing these 7 scores as binary might introduce bias, and we are not sure what each score means, e.g., a 1 in a 4-point Likert score means “not at all,” while 4 means “very much.”*

**Supplementary Table 3 Magnitude of change, showing the percentage of patients that changed and their order of change within minor, moderate, and major change categories**

| Items | Stable, n (%) | Change between not at all and a little, n (%) | | Change between not at all and quite a bit, n (%) | | Change between not at all and very much, n (%) | | Change between a little and quite a bit, n (%) | | Change between a little and very much, n (%) | | Change between quite a bit and very much, n (%) | |
| --- | --- | --- | --- | --- | --- | --- | --- | --- | --- | --- | --- | --- | --- |
|  |  | Not at all – a little  (1 point) | A little – Not at all (1 point) | Not at all -quite a bit (2 points) | Quite a bit - Not at all  (2 points) | Not at all - very much  (3 points) | Very much - Not at all  (3 points) | A little - quite a bit  (1 point) | Quite a bit – A little  (1 point) | A little - very much (2 points) | Very much – a little  (2 points) | Quite a bit - very much  (1 point) | Very much - quite a bit (1 point) |
| PF 1 | 68 (73.9) | 3 (3.3) | 8 (8.7) | 0 (0.0) | 1 (1.1) | 0 (0.0) | 0 (0.0) | 2 (2.2) | 5 (5.4) | 1 (1.1) | 1 (1.1) | 2 (2.2) | 1 (1.1) |
| PF 2 | 72 (78.3) | 6 (6.5) | 1 (1.1) | 0 (0.0) | 0 (0.0) | 0 (0.0) | 0 (0.0) | 3 (3.3) | 4 (4.3) | 0 (0.0) | 0 (0.0) | 2 (2.2) | 4 (4.3) |
| PF 3 | 75 (81.5) | 6 (6.5) | 1 (1.1) | 0 (0.0) | 0 (0.0) | 0 (0.0) | 0 (0.0) | 2 (2.2) | 5 (5.4) | 0 (0.0) | 0 (0.0) | 1 (1.1) | 2 (2.2) |
| PF 4 | 71 (77.2) | 6 (6.5) | 6 (6.5) | 0 (0.0) | 2 (2.2) | 0 (0.0) | 0 (0.0) | 2 (2.2) | 4 (4.3) | 1 (1.1) | 0 (0.0) | 0 (0.0) | 0 (0.0) |
| PF 5 | 85 (92.4) | 2 (2.2) | 1 (1.1) | 0 (0.0) | 1 (1.1) | 0 (0.0) | 0 (0.0) | 1 (1.1) | 2 (2.2) | 0 (0.0) | 0 (0.0) | 0 (0.0) | 0 (0.0) |
| EF1 | 59 (64.1) | 14 (15.2) | 4 (4.3) | 2 (2.2) | 1 (1.1) | 0 (0.0) | 0 (0.0) | 5 (5.5) | 5 (5.4) | 0 (0.0) | 1 (1.1) | 0 (0.0) | 1 (1.1) |
| EF2 | 60 (65.2) | 7 (7.6) | 7 (7.6) | 0 (0.0) | 0 (0.0) | 0 (0.0) | 0 (0.0) | 7 (7.6) | 8 (8.7) | 0 (0.0) | 0 (0.0) | 1 (1.1) | 2 (2.2) |
| EF3 | 56 (60.9) | 11 (12.0) | 11 (12) | 0 (0.0) | 1 (1.1) | 0 (0.0) | 0 (0.0) | 4 (4.3) | 5 (5.4) | 0 (0.0) | 1 (1.1) | 1 (1.1) | 2 (2.2) |
| EF4 | 62 (67.4) | 6 (6.5) | 8 (8.7) | 0 (0.0) | 0 (0.0) | 0 (0.0) | 0 (0.0) | 5 (5.4) | 10 (10.9) | 0 (0.0) | 0 (0.0) | 1 (1.1) | 0 (0.0) |
| RF1 | 56 (60.9) | 5 (5.4) | 10 (10.9) | 1 (1.1) | 1 (1.1) | 0 (0.0) | 1 (1.1) | 3 (3.3) | 8 (8.7) | 0 (0.0) | 2 (2.2) | 2 (2.2) | 3 (3.3) |
| RF2* | 63 (69.2) | 4 (4.4) | 5 (5.5) | 1 (1.1) | 0 (0.0) | 0 (0.0) | 2 (2.2) | 1 (1.1) | 9 (9.9) | 1 (1.1) | 0 (0.0) | 4 (4.4) | 1 (1.1) |
| CF1 | 59 (64.1) | 3 (3.3) | 10 (10.9) | 1 (1.1) | 2 (2.2) | 0 (0.0) | 0 (0.0) | 2 (2.2) | 9 (9.9) | 2 (2.2) | 1 (1.1) | 2 (2.2) | 1 (1.1) |
| CF2 | 59 (64.1) | 7 (7.6) | 7 (7.7) | 0 (0.0) | 2 (2.2) | 0 (0.0) | 0 (0.0) | 5 (5.4) | 4 (4.3) | 2 (2.2) | 0 (0.0) | 0 (0.0) | 6 (6.6) |
| SF1 | 68 (73.9) | 4 (4.3) | 13 (14.1) | 0 (0.0) | 1 (1.1) | 0 (0.0) | 0 (0.0) | 2 (2.2) | 3 (3.3) | 0 (0.0) | 1 (1.1) | 0 (0.0) | 0 (0.0) |
| SF2 | 57 (62.0) | 3 (3.3) | 12 (13.0) | 2 (2.2) | 3 (.3) | 0 (0.0) | 0 (0.0) | 1 (1.1) | 11 (12.0) | 1 (1.1) | 1 (1.1) | 0 (0.0) | 1 (1.1) |
| FA1 | 58 (63.0) | 3 (3.3) | 5 (5.4) | 0 (0.0) | 1 (1.1) | 0 (0.0) | 1 (1.1) | 7 (7.6) | 14 (15.2) | 0 (0.0) | 1 (1.1) | 1 (1.1) | 0 (0.0) |
| FA2 | 59 (64.1) | 8 (8.7) | 11 (12.0) | 0 (0.0) | 1 (1.1) | 0 (0.0) | 1 (1.1) | 2 (2.2) | 10 (10.9) | 0 (0.0) | 0 (0.0) | 0 (0.0) | 0 (0.0) |
| FA3* | 63 (69.2) | 4 (4.3) | 9 (9.9) | 0 (0.0) | 0 (0.0) | 0 (0.0) | 0 (0.0) | 2 (2.2) | 9 (9.9) | 0 (0.0) | 0 (0.0) | 1 (1.1) | 3 (3.3) |
| NV1 | 79 (85.9) | 3 (3.3) | 9 (9.8) | 0 (0.0) | 0 (0.0) | 0 (0.0) | 0 (0.0) | 0 (0.0) | 1 (1.1) | 0 (0.0) | 0 (0.0) | 0 (0.0) | 0 (0.0) |
| NV2 | 89 (96.7) | 2 (2.2) | 0 (0.0) | 0 (0.0) | 0 (0.0) | 0 (0.0) | 0 (0.0) | 0 (0.0) | 1 (1.1) | 0 (0.0) | 0 (0.0) | 0 (0.0) | 0 (0.0) |
| PA1 | 65 (70.7) | 7 (7.6) | 8 (8.7) | 2 (2.2) | 1 (1.1) | 0 (0.0) | 0 (0.0) | 6 (6.5) | 2 (2.2) | 0 (0.0) | 0 (0.0) | 0 (0.0) | 1 (1.1) |
| PA2 | 68 (73.9) | 11 (12.0) | 7 (7.6) | 0 (0.0) | 3 (3.3) | 0 (0.0) | 0 (0.0) | 1 (1.1) | 1 (1.1) | 1 (1.1) | 0 (0.0) | 0 (0.0) | 0 (0.0) |
| DY | 73 (79.3) | 5 (5.4) | 6 (6.5) | 0 (0.0) | 1 (1.1) | 0 (0.0) | 0 (0.0) | 1 (1.1) | 5 (5.4) | 0 (0.0) | 0 (0.0) | 0 (0.0) | 1 (1.1) |
| SL | 67 (72.8) | 5 (5.4) | 7 (7.6) | 1 (1.1) | 0 (0.0) | 0 (0.0) | 0 (0.0) | 5 (5.4) | 6 (6.5) | 0 (0.0) | 0 (0.0) | 0 (0.0) | 1 (1.1) |
| AP | 72 (78.3) | 3 (3.3) | 9 (9.8) | 0 (0.0) | 0 (0.0) | 0 (0.0) | 0 (0.0) | 1 (1.1) | 4 (4.3) | 0 (0.0) | 2 (2.2) | 1 (1.1) | 0 (0.0) |
| CO | 71 (77.2) | 6 (6.5) | 4 (4.3) | 0 (0.0) | 2 (2.2) | 0 (0.0) | 0 (0.0) | 4 (4.3) | 3 (3.3) | 0 (0.0) | 0 (0.0) | 0 (0.0) | 2 (2.2) |
| DI | 81 (89.0) | 4 (4.3) | 3 (3.3) | 1 (1.1) | 1 (1.1) | 0 (0.0) | 0 (0.0) | 0 (0.0) | 0 (0.0) | 1 (1.1) | 0 (0.0) | 0 (0.0) | 0 (0.0) |
| FI | 74 (80.4) | 6 (6.5) | 6 (6.5) | 0 (0.0) | 1 (1.1) | 1 (1.1) | 0 (0.0) | 0 (0.0) | 4 (4.3) | 0 (0.0) | 0 (0.0) | 0 (0.0) | 0 (0.0) |
| FU1* | 67 (73.6) | 4 (4.4) | 7 (7.7) | 0 (0.0) | 0 (0.0) | 0 (0.0) | 0 (0.0) | 3 (3.3) | 6 (6.6) | 0 (0.0) | 0 (0.0) | 2 (2.2) | 2 (2.2) |
| FU2* | 61 (67.0) | 6 (6.6) | 13 (14.2) | 2 (2.2) | 1 (1.1) | 0 (0.0) | 0 (0.0) | 1 (1.1) | 6 (6.6) | 0 (0.0) | 0 (0.0) | 0 (0.0) | 1 (1.1) |
| FU3* | 64 (70.3) | 5 (5.5) | 13 (14.3) | 1 (1.1) | 0 (0.0) | 0 (0.0) | 0 (0.0) | 2 (2.2) | 6 (6.6) | 0 (0.0) | 0 (0.0) | 0 (0.0) | 0 (0.0) |
| FU4* | 63 (69.2) | 5 (5.5) | 10 (11.0) | 0 (0.0) | 1 (1.1) | 0 (0.0) | 0 (0.0) | 3 (3.3) | 6 (6.5) | 0 (0.0) | 0 (0.0) | 2 (2.2) | 1 (1.1) |
| HA | 69 (75.8) | 5 (5.5) | 8 (8.8) | 2 (2.2) | 2 (2.2) | 0 (0.0) | 0 (0.0) | 1 (1.1) | 2 (2.2) | 0 (0.0) | 1 (1.1) | 0 (0.0) | 1 (1.1) |
| VD1** | 84 (93.3) | 1 (1.1) | 5 (5.6) | 0 (0.0) | 0 (0.0) | 0 (0.0) | 0 (0.0) | 0 (0.0) | 0 (0.0) | 0 (0.0) | 0 (0.0) | 0 (0.0) | 0 (0.0) |
| VD2* | 69 (75.8) | 9 (9.9) | 9 (9.9) | 0 (0.0) | 0 (0.0) | 0 (0.0) | 0 (0.0) | 0 (0.0) | 4 (4.4) | 0 (0.0) | 0 (0.0) | 0 (0.0) | 0 (0.0) |
| VD3* | 65 (71.4) | 9 (9.9) | 9 (9.9) | 2 (2.2) | 0 (0.0) | 0 (0.0) | 0 (0.0) | 2 (2.2) | 3 (3.3) | 0 (0.0) | 0 (0.0) | 0 (0.0) | 1 (1.1) |
| SE* | 83 (91.2) | 3 (3.3) | 1 (1.1) | 1 (1.1) | 1 (1.1) | 0 (0.0) | 1 (1.1) | 0 (0.0) | 1 (1.1) | 0 (0.0) | 0 (0.0) | 0 (0.0) | 0 (0.0) |
| MD1* | 80 (87.9) | 2 (2.2) | 3 (3.3) | 2 (2.2) | 1 (1.1) | 0 (0.0) | 0 (0.0) | 1 (1.1) | 1 (1.1) | 0 (0.0) | 0 (0.0) | 0 (0.0) | 1 (1.1) |
| MD2* | 60 (65.9) | 8 (8.8) | 6 (6.6) | 3 (3.3) | 2 (2.2) | 2 (2.2) | 0 (0.0) | 3 (3.3) | 5 (5.5) | 0 (0.0) | 0 (0.0) | 1 (1.1) | 1 (1.1) |
| MD3* | 66 (72.5) | 11 (12.1) | 7 (7.7) | 0 (0.0) | 1 (1.1) | 0 (0.0) | 0 (0.0) | 4 (4.4) | 0 (0.0) | 0 (0.0) | 0 (0.0) | 2 (2.2) | 0 (0.0) |
| CD1* | 69 (75.8) | 4 (4.4) | 3 (3.3) | 0 (0.0) | 0 (0.0) | 0 (0.0) | 0 (0.0) | 2 (2.2) | 6 (6.6) | 0 (0.0) | 3 (3.3) | 1 (1.1) | 3 (3.3) |
| CD2* | 76 (83.5) | 5 (5.5) | 3 (3.3) | 2 (2.2) | 1 (1.1) | 0 (0.0) | 0 (0.0) | 1 (1.1) | 0 (0.0) | 0 (0.0) | 1 (1.1) | 0 (0.0) | 2 (2.2) |
| CD3* | 63 (69.2) | 11 (12.1) | 5 (5.5) | 0 (0.0) | 1 (1.1) | 0 (0.0) | 1 (1.1) | 3 (3.3) | 5 (5.5) | 1 (1.1) | 1 (1.1) | 0 (0.0) | 0 (0.0) |
| DR* | 63 (69.2) | 6 (6.6) | 11 (12.1) | 0 (0.0) | 0 (0.0) | 1 (1.1) | 0 (0.0) | 2 (2.2) | 1 (1.1) | 1 (1.1) | 0 (0.0) | 0 (0.0) | 0 (0.0) |
| HL* | 79 (86.8) | 2 (2.2) | 2 (2.2) | 1 (1.1) | 1 (1.1) | 1 (1.1) | 0 (0.0) | 1 (1.1) | 2 (2.2) | 1 (1.1) | 0 (0.0) | 0 (0.0) | 1 (1.1) |
| IS* | 79 (86.8) | 6 (6.6) | 0 (0.0) | 0 (0.0) | 1 (1.1) | 0 (0.0) | 1 (1.1) | 1 (1.1) | 1 (1.1) | 0 (0.0) | 0 (0.0) | 0 (0.0) | 1 (1.1) |
| WL* | 75 (82.4) | 6 (6.6) | 5 (5.5) | 1 (1.1) | 1 (1.1) | 0 (0.0) | 0 (0.0) | 3 (3.3) | 0 (0.0) | 0 (0.0) | 0 (0.0) | 0 (0.0) | 0 (0.0) |
| BC** | 79 (87.8) | 2 (2.2) | 4 (4.4) | 0 (0.0) | 2 (2.2) | 0 (0.0) | 0 (0.0) | 0 (0.0) | 3 (33.3) | 0 (0.0) | 0 (0.0) | 0 (0.0) | 0 (0.0) |

*Abbreviations: Physical functioning: PF, Role functioning: RF, Emotional functioning: EF, Cognitive functioning: CF, Social functioning: SF, Fatigue: FA, Nausea and vomiting: NV, Pain: PA, Dyspnea: DY, Sleep: SL, Appetite loss: AP, Constipation: CO, Diarrhea*: DI, Financial difficulties: FI, Future uncertainty*: FU, Visual deficits*: VD, Motor dysfunction*: MD, Communication deficit*: CD, Headache*: HA, Seizures: SE*, Drowsiness*: DR, Hair loss*: HL, Itchy skin*: IS, Weakness of the leg*: WL, Bladder control**: BC. Available case analysis was performed with * a total number of patients=91 and ** total number of patients=90.*
